# Chimeric galectin-3 and collagens: Biomarkers and potential therapeutic targets in fibroproliferative diseases

**DOI:** 10.1016/j.jbc.2022.102622

**Published:** 2022-10-20

**Authors:** Pratima Nangia-Makker, Victor Hogan, Vitaly Balan, Avraham Raz

**Affiliations:** 1 Barbara Ann Karmanos Cancer Institute, Department of Oncology, School of Medicine, Redwood City, California, USA; 2 Guardant Health, Bioinformatics, Redwood City, California, USA; 3 Department of Pathology, School of Medicine, Wayne State University, Detroit, Michigan, USA

**Keywords:** galectin-3, collagens, fibroproliferative diseases, myocardial fibrosis, hepatic fibrosis, pulmonary fibrosis, biomarkers, CITP, C-terminal fragment of collagen type I degradation, COPD, chronic obstructive pulmonary disease, ECM, extracellular matrix, ILD, interstitial lung disease, IPF, idiopathic pulmonary fibrosis, MCP, modified citrus pectin, MMP, matrix metalloprotease, NASH, nonalcoholic steatohepatitis, ND, N-terminus domain, PAH, Pulmonary arterial hypertension, PICP, C-terminal propeptide of procollagen type I, PINP, procollagen type I N-terminal propeptide, SSc, systemic sclerosis, TGF-β, transforming growth factor-β

## Abstract

Fibrosis, stiffening and scarring of an organ/tissue due to genetic abnormalities, environmental factors, infection, and/or injury, is responsible for > 40% of all deaths in the industrialized world, and to date, there is no cure for it despite extensive research and numerous clinical trials. Several biomarkers have been identified, but no effective therapeutic targets are available. Human galectin-3 is a chimeric gene product formed by the fusion of the internal domain of the collagen alpha gene [N-terminal domain (ND)] at the 5′-end of galectin-1 [C-terminal domain (CRD)] that appeared during evolution together with vertebrates. Due to the overlapping structural similarities between collagen and galectin-3 and their shared susceptibility to cleavage by matrix metalloproteases to generate circulating collagen-like peptides, this review will discuss present knowledge on the role of collagen and galectin-3 as biomarkers of fibrosis. We will also highlight the need for transformative approaches targeting both the ND and CRD domains of galectin-3, since glycoconjugate binding by the CRD is triggered by ND-mediated oligomerization and the therapies targeted only at the CRD have so far achieved limited success.

The extracellular matrix (ECM) is the noncellular scaffold structure present within all tissues and organs, composed mainly of proteoglycans and fibrous proteins. It is crucial for tissue morphogenesis, differentiation, and homeostasis. ECM can be divided into basement membrane and interstitial matrix. The basement membrane, which functions as a scaffold for epithelial and endothelial cells, is composed of collagen type IV, laminin, nidogen (enactin), and perlecan. The major component is collagen type IV, constituting about 50% of all basement membrane proteins. Laminin is the major noncollagenous component of the basement membrane. The interstitial matrix, mainly produced by fibroblasts and composed of collagen I, III, V, and VI, fibronectin, and proteoglycans makes up the majority of ECM in the body. These components together assemble in a highly cross-linked network, with great functional and compositional variations allowing rapid diffusion of certain small molecules (reviewed in (1)).

Tissue injury knocking out epithelial and endothelial cells and exposure of basement membrane results in an influx of inflammatory cells, such as macrophages and neutrophils into the damaged site. These cells secrete proteases to degrade the basement membrane and release the fragments of component proteins into circulation. To repair the basement membrane, the activated fibroblasts secrete new proteins to substitute the degraded proteins (Fig. 1*A*). This results in wound healing. In case of chronic inflammation, the deeper tissues including the interstitial layer of ECM are exposed and get damaged. Several inflammatory cytokines including the interleukins (2, 3) and members of transforming growth factor-β (TGF-β) (4, 5) secreted by platelets, endothelial cells, smooth muscle cells, and macrophages act on fibroblasts to induce proliferation and differentiation. The differentiated fibroblasts continue to secrete proteins leading to disproportionate accumulation of collagen in the interstitial space, causing scarring of the affected organ (1) (Fig. 1*B*). Galectin-3 is a profibrotic molecule regulating the functions of macrophages and fibroblasts in response to inflammation. It was shown that increased galectin-3 expression further activates myofibroblasts leading to wound scarring and is thus implicated in inflamed organ’s ‘fibrosis’(Fig. 1). On the other hand, its deficiency leads to reduced fibrotic response.

**Figure 1:**
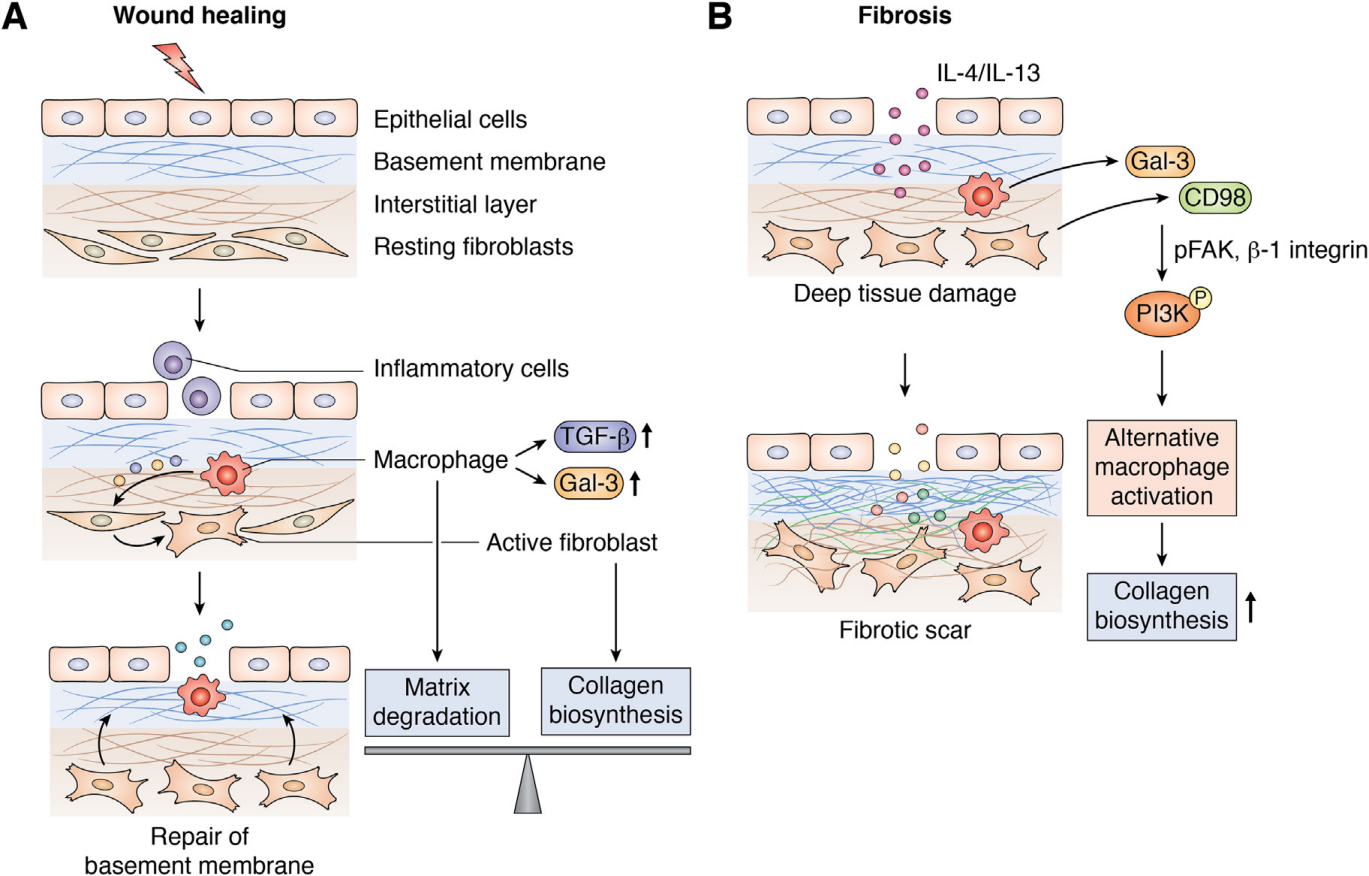
**Schematic presentation of wound healing and fibrosis**: (*A*) Cell death resulting from tissue injury results in recruitment of inflammatory cells through the damaged epithelium. These cells secrete proteases, degrade basement membrane, and release the components into circulation. Galectin-3 and TGF-β secreted by macrophages activate the resting fibroblasts into myofibroblasts, which secrete fresh basement membrane resulting in wound healing. The synthetic and degradative processes are in balance. (*B*) Constant and repetitive injury induces damage to the deeper interstitial layer and results in overproduction of the basement membrane components in an unorganized way leading to fibrosis. Secretion of IL-4 and IL-13 by inflammatory cells activates alternative macrophages, activated macrophages secrete increased galectin-3 and overexpress its cell surface receptor CD98. Galectin-3 and CD98 binding stabilizes CD98 and activates PI3K *via* an association with phosphorylated FAK and β-1 integrin. A galectin-3 feedback loop drives alternative macrophage activation. (Adapted from Genovese and Karsdal, 2016, Expert Review of Proteomics, 13 (2), 213–225).

The abnormal remodeling of the ECM is related to a plethora of fibroproliferative diseases of various organs including heart, liver, lung, kidneys, skin, and some systemic disorders such as systemic sclerosis, atherosclerosis, and cystic fibrosis (Fig. 2) and is responsible for nearly 45% of all deaths (6). The mechanism of fibrosis is similar in various organs as all epithelial tissues such as skin, digestive tract, pulmonary organs, genitourinary tracts, and endothelial cells of blood vessels, as well as mesothelial cells in the body cavities, are lined by ECM (7, 8). In recent years, fibroproliferative process has been the focus of interest as a candidate for disease intervention. The noninvasive method of detecting fibrosis is by analyzing the levels of circulating biomarkers. Several biomarkers have been studied including collagen-related peptides, matrix metalloproteases (MMPs), tissue inhibitors of metalloproteases, selected miRNAs, galectin-3, and several noncollagen-related peptides. Among these, collagen peptides and galectin-3 appear to be most promising as reflecting combined effects of injury, inflammation, and fibrosis. In this review, we have selected the liver, heart, and lung as representative organs of fibroproliferative diseases, and we will discuss the current knowledge on these two proteins and their peptides as biomarkers of fibrosis and disease progression and value of galectin-3 as a therapeutic target.

**Figure 2:**
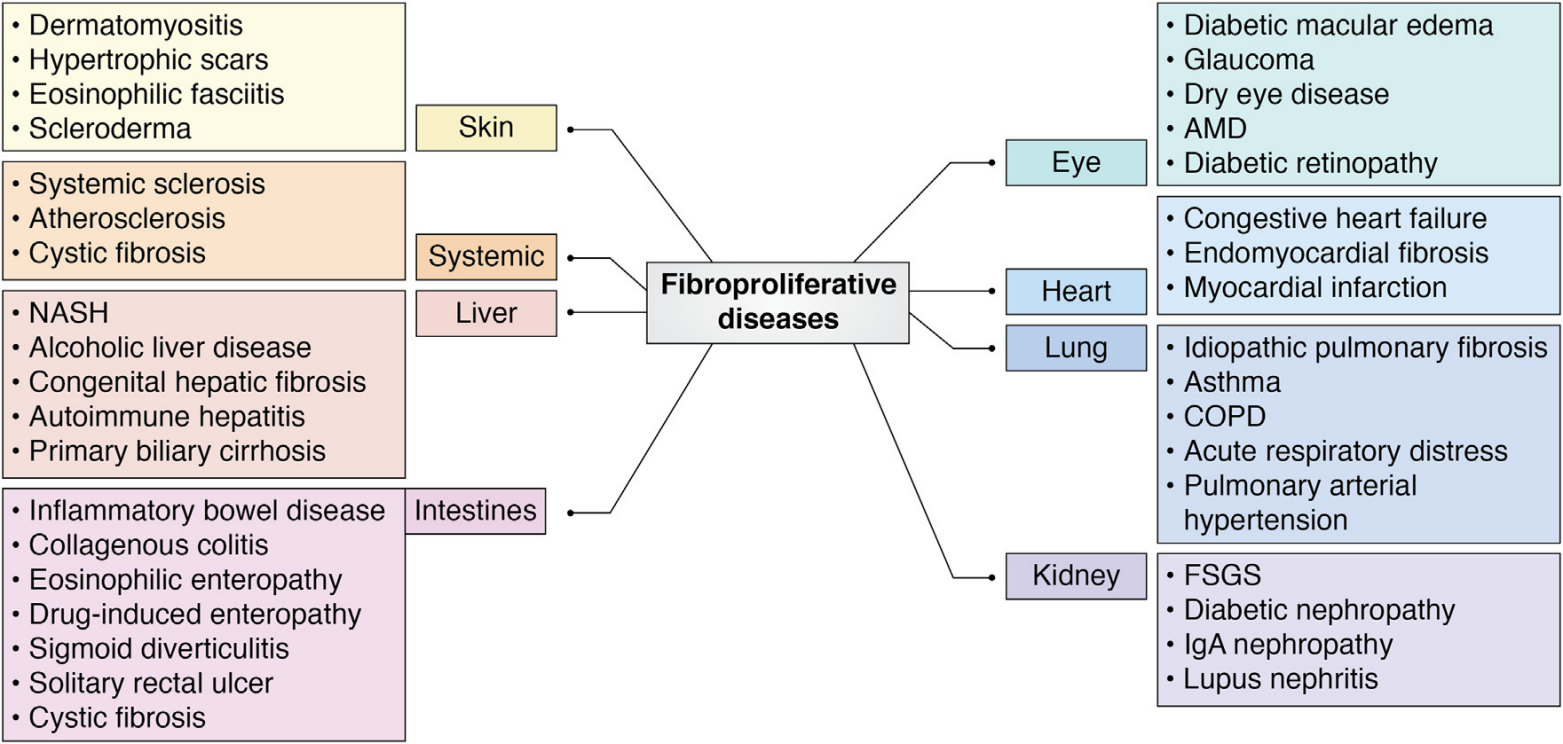
**Fibroproliferative diseases: Various organs develop fibrotic scars as a result of constant tissue damage and insults**. FSGS, focal segmental glomerlosclerosis; NASH, nonalcoholic steatohepatitis; AMD, age-related macular degeneration; COPD, chronic obstructive pulmonary disease. (Adapted from Karsdal *et al*, 2014 Alimentary Pharmacology and Therapeutics, 40: 233–249)

## Biomarkers of fibrosis

### Collagens

Collagens are a superfamily of 28 members comprising the most abundant proteins in humans. The common feature of all family members is a triple helix structure made up of three polypeptide chains, which can either be homotrimers (3 identical α chains) or heterotrimers (nonidentical α chains) and are of variable lengths in different members. Collagens are characterized by their capacity to form supramolecular assemblies (9). Based on the supramolecular structure, the collagens can be fibrils, beaded filaments, anchoring fibrils, and networks (10). Further diversity in the collagen family is due to the several molecular isoforms as well as alternative splicing and alternative promoters. Collagens are synthesized as procollagens and cleaved to mature form. The mature collagens are enzymatically cleaved and released as biologically active fragments (10). As collagens are an integral part of the ECM, their deregulated cleavage and reassembly play an important role in fibrosis. Circulating collagen fragments (neoepitopes) as biomarkers of the fibrogenic or fibrolytic events in various diseases have been studied extensively. Propeptides, which are released from procollagen as part of the maturing process, reflect the synthetic process, whereas the degradation epitopes, which are released as part of the degradation process reflect the fibrolytic process (9). Various collagen epitopes used as biomarkers have been summarized in Table 1.

**Table 1:** Major collagen neo-epitopes used as serum biomarkers for fibroproliferative diseases

Synthesis-related epitopes			
Collagen type	Name	Description	Affected organ
Collagen type I	PINP	Amino-terminal peptide of procollagen type I	Heart
	PICP	C-terminal peptide of procollagen type I	Heart
Collagen type III	PIIINP	Amino-terminal peptide of procollagen Type III	Liver, heart, and lung
	Pro C3	A fragment of N-terminal type III collagen	Lung
Collagen type IV	P4NP7	7S domain of type IV collagen	Liver
	NC-1	Carboxy-terminal region of alpha chain	Liver
Collagen type VI	ProC6	A fragment of C-terminal type VIa3 collagen	Lung


Degradation-related epitopes			
Collagen type	Name	Description	Affected organ
Collagen type I	CIM	MMP degraded fragment of Collagen type I	Lung
	CITP	Carboxy terminal telopeptide of collagen I	Heart
Collagen type III	C3M	MMP degraded fragment of Type III collagen	Liver, Lung
	C3A	ADAMTS degraded fragment of type III collagen	Lung
Collagen type IV	C4M	MMP degraded fragment of type IV collagen	Liver
Collagen type V	C5M	MMP degraded fragment of type V collagen	Lung
Collagen type VI	C6M	MMP degraded fragment of type VI collagen	Lung

### Galectin-3

Human galectin-3 (LGALS3), a protein of 31Kda (11, 12), belongs to the galectin gene family of carbohydrate-binding proteins and is the only member that is expressed in vertebrates. All of the galectin family members contain a conserved carbohydrate-binding domain of ∼130 amino acids. Galectins have been divided into three subtypes based on their structure: prototype, tandem repeat, and chimera. Galectin-3 is the only chimera (fused) protein consisting of three distinct structural motifs: a short 12 amino acid N-terminal motif, followed by a long collagen α-like sequence (collagen α) and a C-terminal carbohydrate-binding domain (galectin-1).

**Figure 3:**
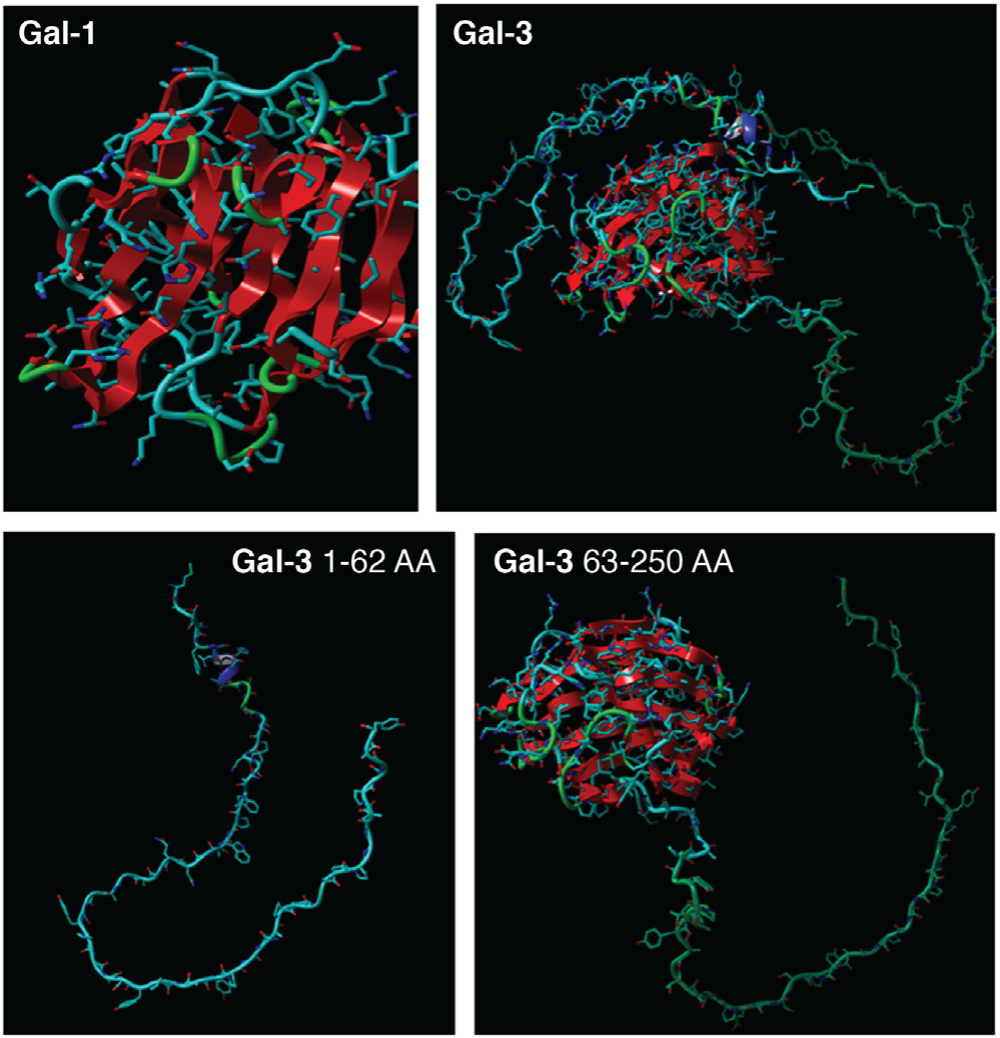
**Molecular structure of galectin-1(top left) and galectin-3 (top right**). *Lower pane*l: cleaved galectin-3 N-terminal and C-terminal. Visualization and analysis was done by YASARA model software.

The short N-terminal motif contains a site of serine^6^ phosphorylation for controlling the nuclear transport and ligand affinity (13). The long and intrinsically disorganized collagen α like proline-rich sequence of about 110 amino acids (ND) is cleavable by MMPs -2, -9, and membrane type 1 MMP at the Ala^62^-Tyr^63^ bond, resulting in the generation of a cleaved fragment of 22kD (14) (Fig. 3). The C-terminal domain (CRD), consisting of about 130 amino acids, is the common domain shared by all galectin family members and is responsible for their lectin activity. Galectin-3 has a preferential binding for N-acetyllactosamine residues on cell surface glycoconjugates. Galectin-3 interacts with several ligands both intracellularly and extracellularly and influences various pathways and processes *via* its CRD binding activity. It binds Bcl-2, CD95, Nucling, Alix/AIP1, synexin, and regulates apoptosis. Its interaction with activated K-Ras protein affects cell proliferation and survival. β-catenin is its binding partner in the Wnt signaling pathway. Galectin-3 binds to laminin, fibronectin, hensin, elastin, collagen IV, and tenascin-C and tenascin-R to modulate cell–ECM adhesion. In addition, it also binds α1β1, αvβ3, and αMβ1 integrins, the main proteins involved in cell adhesion (Table 2).

A germ-line mutation at position 191 (rs4644) substituting amino acid proline^64^ to histidine makes this protein susceptible to MMP cleavage and enhances its migratory and angiogenic potential (15, 16). It was reported that cleaved galectin-3 had stronger affinity for glycoconjugates than the full-length protein (17), while some other interactions require both N-terminus domain and CRD motifs. Galectin-3 displays multivalency by the hydrophobic interactions of the N terminal with itself and with the CRD forming a fuzzy complex, which is the characteristic of intrinsically disordered proteins to achieve liquid–liquid phase separation (18). Additional functional oligomeric states exist due to the dynamic homodimerization of the N terminal. It is generally accepted that galectin-3 oligomerization gives rise to changes in activity, which are associated with and reflected in its diverse biological functions. Galectin-3 oligomer forms a lattice with T cell surface receptors that prevents their uncontrolled activation (19), and its cross-linking with either EGF and TGF-β receptors delays their internalization and degradation (20). Table 2 reflects the several biological processes regulated by C-terminal and N-terminal domains of galectin-3 in association with various binding partners.

**Table 2:** Binding partners of galectin-3 C-terminal and N-terminal

C-terminal	Function	Reference
Bcl2	Apoptosis	Yang *et al*, 1996 (145)
CD95	Apoptosis	Fukumori *et al*, 2004 (146)
Nucling	Apoptosis	Liu *et al*, 2004 (147)
Alix/AIP1	Apoptosis	Liu *et al*, 2002 (148)
K-Ras	Cell proliferation	Eelad-sfadia *et al*, 2004 (149)
Akt	Cell Proliferation	Lee *et al*, 2003 (150), Oka *et al*, 2005 (151)
β-catenin	Wnt signaling	Shimura *et al*, 2004 (152)
Laminin	ECM adhesion	Massa *et al*, 1993 (153),
Fibronectin	ECM adhesion	Sato et al, 1992 (154)
Hensin	ECM adhesion	Hikita *et al*, 2000 (155)
Elastin	ECM adhesion	Ochieng *et al*, 1998 (156)
Collagen IV	ECM adhesion	Ochieng *et al*, 1998 (157)
Tenascin-C&-R	ECM adhesion	Probstmeier *et al*, 1995 (158))
α1β1 integrin	Cell adhesion	Ochieng *et al*, 1998 (157)
CD11b/CD18	Inflammatory macrophage	Dong *et al*, 1997 (28)
Lamp-1 and -2	Inflammatory macrophage	Dong *et al*, 1997 (28)
IgE	Inflammation	Cherayil *et al*, 1989 (159)
CD44	Cargo protein internalization	Lakshminarayan *et al*, 2014 (160)
CD98	Membrane trafficking	Dalton *et al*, 2007 (161)
CD66	Inflammatory neutrophils	Feuk-Lagersted *et al*,1999 (162)
N-terminal	Function	Reference
EGFR	Cross-linking & endocytosis	Partridge *et al*, 2004 (163); Liu 2012 (164)
TGFβR	Cross linking& endocytosis	Partridge *et al*, 2004 (163)
CD147	Clustering &MMP9 induction	Mauris *et al*, 2014 (138)
Alix	HIV infection	Wang *et al*, 2014 (139)
Alix	T cell receptor (TCR) downregulation	Chen *et al*, 2009 (165)
Bacterial LPS	Inflammation	Lo *et al*, 2021 (142)

Abbreviations: Alix, ALG2 interacting protein X; CEA, carcinoembryonic antigen.

Galectin-3 is a profibrotic molecule and implicated in modulation of fibroblasts and macrophage activity in chronically inflamed lung, liver, kidney, heart, skin, blood vessels, etc. affecting common fibroproliferative pathways leading to fibrosis (21, 22). It is a proinflammatory molecule (23). It regulates immune functions and mediates acute and chronic inflammation. It activates and is abundantly expressed in cells of myeloid origin, such as monocytes, macrophages, dendritic cells, and neutrophils (24). It interacts with inflammatory cytokines TGF-β and CD98 expressed by migrating inflammatory cells and plays a major role in the profibrotic response (5). In galectin-3–deficient mice, a dramatic reduction in fibrosis in response to TGF-β and bleomycin was observed accompanied with reduced epithelial to mesenchymal transition and myofibroblast activation (25, 26). Galectin-3 is instrumental in TGF-β1–induced fibroblasts differentiation *via* the MAPK/extracellular signal-regulated kinase (ERK)-ERK 1/2 signaling pathway (Fig. 1). It aids the extravasation of inflammatory cells and binds to specific cell surface receptors on macrophages (CD11b, CD98) and on neutrophils (CD66). It is upregulated in alternative macrophage activation by IL4 and IL13, and it activates PI3K *via* binding to CD98 and aids in increased collagen deposition (27, 28) by differentiation of resting fibroblasts into myofibroblasts leading to scar formation (29) (Fig. 1).

## Hepatic fibrosis

Hepatitis B and Hepatitis C virus infections, innate immunity, and chronic inflammation play a role in the pathogenesis of liver metabolic disorders, nonalcoholic fatty liver disease, liver steatosis, nonalcoholic steatohepatitis (NASH), fibrosis, and cirrhosis. Chronic liver disease and cirrhosis result in ∼35,000 deaths each year in the US and for approximately two million deaths per year worldwide.

The major histological components of the liver consist of (i) hepatocytes, constituting the parenchyma, (ii) stroma, (iii) sinusoids (the capillaries travelling between hepatocytes), and (iv) the spaces of Disse, which are located between the hepatocytes and the sinusoids. The liver sinusoids are a unique structure and are classified as discontinuous capillaries as they do not have a continuous endothelial lining but are endowed with fenestrated endothelium, which enables liver functions like ultrafiltration, endocytosis, and immunological activities. The spaces of Disse is enriched in collagen IV and perlecan but lacks laminin and nidogen (other components of the basement membrane). Sinusoidal capillarization is characterized by the formation of the basal lamina, loss of fenestrae, and transformation of the sinusoids into the continuous capillary. This interferes with hepatic microcirculation and leads to hepatic dysfunction (30). The capillarization of sinusoids is the basic remodeling in all chronic liver diseases, accompanied with increased collagen IV and laminin deposits in the spaces of Disse.

### Collagen fragments as biomarkers of hepatic fibrosis

Collagen IV expression levels were used to distinguish between the early and late stages of fibrosis in hepatitis C-related fibrosis (31). A 14-fold increase in collagen IV was observed in liver cirrhosis (32), and the levels were higher in alcoholic than in nonalcoholic hepatitis and cirrhosis (33, 34). In addition, neoepitopes of collagen IV: 7S and NCI (noncollagenous C-terminal domain of collagen IV) domains have been studied as noninvasive biomarkers of chronic liver disease. A correlation between the serum 7S collagen fragment levels and liver fibrosis grade was observed in chronic hepatitis C (35), and these levels were much higher in chronic hepatitis C with cirrhosis (36, 37). A similar relationship was observed with NCI and progressive liver disease cirrhosis, when compared with chronic active hepatitis (38, 39). To date, the most used marker of liver fibrosis is the amino-terminal peptide of procollagen type III (PIIINP) (40, 41, 42) together with C3M, which is an MMP degraded fragment of type III collagen reported to be elevated in liver fibrosis, skin fibrosis, and ankylosing spondylitis (43, 44, 45).

### Galectin-3 as a biomarker of hepatic fibrosis

Galectin-3–null mice fed high-fat diet exhibited all the symptoms of nonalcoholic fatty liver disease including increased liver weight, elevated triglycerides, hyperglycemia, and hepatic steatosis, as well as inflammation and fibrosis (44) subsequently developing liver nodules progressing to hepatocellular carcinoma (44, 45). However, some other investigators have documented protection from NASH with attenuation of fibrosis, inflammation, and hepatic injury and other symptoms related to high-fat diets such as hepatocyte degeneration and focal necrosis in galectin-3 KO mice (46, 47). In the carbon tetrachloride-induced cirrhosis mouse model, galectin-3 played a role in ECM production (48) and was responsible for diet-induced steatohepatitis through IL-33/ST2 axis (48).

The removal of advanced glycation end products and advanced lipidomic end products is performed by the liver and galectin-3 is reported to be directly involved with the endocytosis of these harmful byproducts by the liver's sinusoidal and endothelial cells. In galectin-3–null mice, the circulating levels of advanced glycation end products/advanced lipidomic end products were higher than the control mice (46, 47), though overexpression of galectin-3 is related to detoxification protection (48).

## Myocardial fibrosis

Cardiac fibrosis is a significant worldwide health problem associated with nearly all forms of heart disease causing >650,000 deaths in the US. Formation of a fibrotic scar in the cardiac muscle is a fundamental part of several cardiovascular diseases, such as heart failure (HF), dilated cardiomyopathy, and hypertrophic cardiomyopathy and is characterized by activation and differentiation of cardiac fibroblasts into myofibroblasts leading to increased matrix stiffness and abnormalities in cardiac function (for review see (49)).

### Collagen fragments as biomarkers of myocardial fibrosis

Both the synthesis and breakdown-related neoepitopes of collagen have been studied as biomarkers of cardiac fibrosis. The major component of the cardiac ECM is collagen type I, which makes fibrillar structures in combination with collagen type III. In addition, collagen type IV, V, and VI are also present in small amounts in the pericellular space and around the myocytes (50, 51, 52). Plasma levels of C-terminal propeptide of procollagen type I (PICP), which is released as a byproduct of the maturing process of procollagen type I, were shown to be correlated with the myocardial PICP content and collagen volume fraction as determined histologically in hypertrophic cardiomyopathy (53) and hypertension patients (54). However, in a study on the heart failure model in rats, no such correlation was observed (55) indicating the involvement of other confounding factors such as weight loss and a catabolic state. In a study including 111 patients with decompensated heart failure, serum PICP levels were found to be significantly increased in patients that underwent new hospitalizations or death (56). These authors concluded that PICP could be used as an independent predictive biomarker of heart failure, hospitalization, and death.

Another synthesis biomarker of collagen type I is the procollagen type I N-terminal propeptide (PINP), a cleavage product by the proteolytic activity of the ADAMTS family (57). A few studies have examined its validity as a biomarker for fibrosis. Zile *et al.* in a large case-control study reported that in patients of heart failure with reduced ejection fraction, levels of PINP and PIIINP were higher than the controls (58). In a rat model of ischemic cardiomyopathy, plasma PINP levels were much higher than the controls (59). Various studies have shown that serum PIIINP levels can be used as a biomarker of myocardial fibrosis. PIIINP levels in the serum were higher and could be correlated to the cardiac collagen III levels in patients suffering from idiopathic or ischemic dilated cardiomyopathy (60). Higher serum PIIINP levels could also be related to advanced heart diseases (61).

MMPs are the matrix metalloproteases that degrade mature collagen into smaller biologically active fragments. A C-terminal fragment of collagen type I degradation (CITP) has been of interest as a biomarker of fibrolysis. There are some controversial data on the relationship of CITP to heart disease. Lombardi *et al.* in a cross-sectional study demonstrated that levels of CITP were higher, while the levels of fibrogenic markers of collagen type I (PICP and PINP) were not affected indicating a shift toward the breakdown of collagen type I in hypertrophic cardiomyopathy patients (62). Similarly, elevated serum CITP levels were observed in patients with heart failure and atrial fibrillation (63, 64). Circulating levels of CITP were considered as an independent prognostic biomarker of acute myocardial infarction–related death (65). However, another study did not find a correlation of circulating CITP levels with either cardiac collagen type I and III expression levels or with other left ventricle remodeling parameters (66). Ding *et al.* (61) stated that CITP levels may have a diagnostic and prognostic value in myocardial fibrosis.

### Galectin-3 as a biomarker of myocardial fibrosis

Several cardiovascular diseases, especially those that result from chronic inflammation, have been associated with increased serum galectin-3 levels. In 2017, the American Heart Association recommended that plasma levels of galectin-3 could be used as a risk factor and prognosis biomarker of heart failure (67). A pooled analysis of data from three cohorts (COACH, PRIDE, and UDM H-23258) showed that plasma galectin-3 concentration > 17.8 ng/ml was predictive of rehospitalizations in heart failure patients (68). Several other studies have shown a positive correlation between serum galectin-3 levels and prognosis for heart failure (69, 70, 71); however, some reports did not find such a correlation (72, 73). In their recent review, Blanda *et al.* concluded that galectin-3 has a prognostic value for HF patients, but its value in the prediction of early diagnosis of HF is not so certain (74).

Atherosclerosis, the plaque deposition in the arteries results from several risk factors including hyperlipidemia, hypertension, diabetes, and insulin resistance. It was shown that patients with unstable plaques had higher circulating galectin-3 levels than those with stable plaques (75). Moreover, a correlation was also shown between the number of compromised vessels and serum galectin-3 levels (75). Several other studies have shown higher levels of galectin-3 in advanced carotid atherosclerosis (76, 77) and demonstrated that galectin-3 is an independent biomarker of advanced atherosclerosis independent of age, sex, LDL cholesterol levels, and history of acute myocardial infarction (77). Galectin-3 was reported to directly affect the functioning of the three cell types involved in the development of atherosclerosis, *i.e.*, the dysfunction of endothelial cells, differentiation of monocytes to macrophages and foam cells, and proliferation and migration of vascular smooth muscle cells (reviewed in (78)). Sharma *et al.* (79) demonstrated the conversion of fibroblasts to myofibroblasts by treating the cells with recombinant galectin-3, resulting in the expression of TGF-β, collagen, and cyclin D1. Activation of macrophages by galectin-3 resulted in increased production of IL-4 and IL-13 and ECM deposition (25). Galectin-3 also increases the collagen I synthesis in HL-I cardiomyocytes (80).

Ferreira *et al.* reported that galectin-3 is associated with the onset of left ventricular diastolic dysfunction in postmyocardial infarction patients (81). In a recent study, based on 5805 participants, Mortensen *et al.* (82) concluded that low galectin-3 was a useful independent negative predictor of cardiovascular risk. In a long-term follow-up study, patients with high plasma galectin-3 levels showed higher mortality rates (83, 84), while those without acute events showed decreased galectin-3 levels (85). High serum galectin-3 but not galectin-1 levels were associated with increased incidence of large atherosclerotic stroke (86) and postoperative stroke in patients undergoing carotid endarterectomy, postoperative cerebrovascular ischemic events (87). Galectin-3 distribution was altered in arteries from patients with the peripheral arterial disease; its expression was mainly localized in the middle media layer in peripheral arterial disease patients as compared to the outer adventitia layer in normal arteries (88).

## Pulmonary fibrosis

Pulmonary fibrosis is a severe, lifelong lung disease that is nearly always fatal and affects ∼ 128,000 people per year in the US with recorded early mortality of ∼ 40,000 patients per year. It is manifested as lung scarring and results as a pathological response when the compensatory mechanisms for remodeling normal tissue integrity fail after constant and persistent abuses such as lung infection, cigarette smoking, drug, or radiation treatment. When fibrosis of the lung occurs in response to interstitial lung disease (ILD), it is more serious as it is progressive; the worst prognosis has been reported for idiopathic pulmonary fibrosis (IPF) with a median survival of about 3 years after diagnosis. Pulmonary arterial hypertension (PAH) is common in patients with ILD. It is characterized by thickening of the pulmonary wall resulting in high arterial blood pressure contributing to the failure of the right ventricle. In these patients, fibrosis occurs in both the lung blood vessels as well as in the right ventricle (89).

### Collagen fragments as biomarkers of pulmonary fibrosis

The normal lung tissue contains twice as much type I collagen as type III collagen, with these two being the main collagen types (90, 91). It was reported that in early fibrosis, both collagen levels increase, but the ratio changes in favor of type III collagen. In long-standing fibrotic scars mainly collagen I remained (92, 93, 94), which is less elastic and contributes to fibrosis-related abnormalities. In addition to increased collagen I and its cross-linking, there is also increased elastin, fibronectin, hyaluronan, and proteoglycans all contributing to the inaccessibility of collagen cleavage sites by the proteolytic enzymes (95, 96, 97). Sand *et al.* (98) developed ELISA assays to assess the levels of circulating fragments of type IV collagen α1 and α3 chains as indicators of fibrosis and related their elevated levels to liver fibrosis and IPF or chronic obstructive pulmonary disease (COPD), respectively. Teles-Grilo *et al.* (99) demonstrated an increased expression of collagen type I and IV around the new pulmonary blood vessels of bleomycin-treated rats where pulmonary fibrosis was induced. The levels of neoepitope PIIINP increased significantly in broncho alveolar lavage fluid of IPF patients compared to controls (100). Tzortzaki *et al.* (101) showed expressions of collagen type XII and XIV in the lung’s fibrosis of IPF patients and overexpression of collagen type I in the fibrotic scar. Leeming *et al.* (102) were the first group to investigate the collagen turnover profiles in the serum of patients with lung fibrotic disease. They demonstrated that MMP-mediated degradation products of collagen type I, III, V, and VI (CIM, C3M, C5M, C6M) levels could differentiate between IPF and mild COPD patients and healthy controls. Elevated C4M and C3A (neo-epitope of ADAMTS-4, 5–mediated degradation of type III collagen) levels could be related to COPD.

Su *et al.* (103) showed that serum levels of PIIINP and three other ECM molecules (laminin, collagen type IV, and hyaluronic acid) could be used as biomarkers of disease progression from IPF to acute exacerbation of IPF and connective tissue disease–related ILD. Circulating levels of PIIINP were also reported to be higher in patients with progressive pulmonary fibrosis but not in those with stable fibrosis (104). Kubo *et al.* (105) analyzed nine serological biomarkers of collagen metabolism and organ involvement in 79 patients with systemic sclerosis (SSc) and concluded that increased turnover of collagen seen in patients of SSc may not be derived only from the skin. They reported that SSc patients with ILD or PAH showed increased type VI collagen metabolism as indicated by increased levels of C6M. In addition, ProC3 (a fragment of N-terminal type III collagen) and ProC6 (a fragment of C-terminal type VI a3 collagen) were also higher in SSc patients with PAH. In a prospective cohort study, 11 neoepitopes of MMP-degraded ECM proteins were tested in participants with idiopathic pulmonary fibrosis or idiopathic nonspecific interstitial pneumonia. In patients with progressive idiopathy pulmonary fibrosis, serum levels of C1M, C3A, C3M, C6M, CRPM (C-reactive protein metabolite), and VICM (a fragment of vimentin released by MMP) were significantly higher compared to healthy controls (106).

Lung tissues of the patients with IPF showed higher expression of collagen type VI α1 and collagen type VI α3 chains in the fibrotic foci containing a myofibroblasts core and procollagen I (107, 108). In addition, collagen type VI also interacts with ECM and cell surface proteins and forms a filamentous mesh around collagen I, II, III, and IV fibers (109). It was suggested that collagen type VI may also have a regulatory role in the early events of pulmonary fibrosis (110).

### Galectin-3 as a biomarker in pulmonary fibrosis

Calvier *et al.* demonstrated a direct correlation between the onset of vascular hypertrophy, inflammation, and fibrosis with galectin-3 levels (111). Galectin-3 affects STAT 3 and MMP 9 signaling pathways in response to TGF-β induction and mediates vascular fibrosis (112). In a mouse model, TGF-β and bleomycin-induced lung fibrosis was blocked by TD139, a galectin-3 small molecule inhibitor with an affinity for carbohydrate-binding domain (26) *via* inhibiting β-catenin nuclear translocation. Similar effects were replicated in galectin-3 knockout mice (26).

In patients with PAH, serum galectin-3 levels were higher than the base levels (113) while other studies showed that serum galectin-3 could be a biomarker of the severity of PAH (114, 115). Feng *et al.* (116) demonstrated an increase in serum galectin-3 levels in patients with acute exacerbation of chronic obstructive pulmonary disease. Furthermore, the levels of circulating galectin-3 were higher in smokers compared to nonsmokers with acute exacerbation of chronic obstructive pulmonary disease. Idiopathic inflammatory myopathy patients with associated ILD showed elevated levels of serum galectin-3 compared to the healthy controls accompanied by increased galectin-3 expression in the inflammatory cells of interstitial fibrosis, myositis, and dermatitis (117). In a recent study, d’Alessandro *et al.* (118) reported a significant increase in serum galectin-1 and galectin-9 levels in fibrotic ILD patients compared to healthy controls.

## Galectin-3 as a therapeutic target in fibroproliferative diseases

Galectin-3 secreted by macrophages, epithelial cells, and myofibroblasts regulates fibrosis, resulting in pathophysiological responses that include epithelial mesenchymal transition, apoptosis, activation, and proliferation of myofibroblasts resulting in increased production of ECM. Out of all the galectin family members, galectin-3 has shown the strongest involvement with fibrosis. Several preclinical and clinical studies have been conducted aiming at galectin-3 as an antifibrosis target; however, the results were not consistent.

Modified citrus pectin (MCP) was first reported as a functional inhibitor of galectin-3 (119, 120, 121). Later, other complex polysaccharides, *e.g.*, belapectin, GCS-100, pectasol, and several small molecule inhibitors GM-CT-01, GR-MD-02, TD139, and GB1211, all focused on carbohydrate-binding domain, became commercially available as fibrosis inhibitors for preclinical and clinical studies. In galectin-3 null mice, hypoxia-induced right ventricle hypertrophy was ameliorated (122). Similar results were observed in N-Lac (a nonspecific galectin-3 inhibitor) ---treated mice (123). Barman *et al.* demonstrated a direct correlation between the galectin-3 levels and the pulmonary vascular remodeling using galectin-3 KO mice or using GM-CT-01 and GR-MD-02 inhibitors using an animal model of PAH (124).

Inhalation of a small molecule galectin-3 inhibitor TD139, having a strong affinity to the carbohydrate-binding domain, has been shown to reduce galectin-3 expression in the lungs of idiopathic pulmonary fibrosis patients along with decreased circulating levels of platelet-derived growth factor-BB, plasminogen activator inhibitor-1, galectin-3, CCL18, and YKL-40 (125). Another small oral molecule GB1211 is under development but has not been tested for fibrosis inhibition. GR-MD-02 attenuated the NASH-related hepatic fibrosis in a thioacetamide-induced mouse model of liver fibrosis and has undergone clinical trials for the treatment of NASH with advanced fibrosis (126, 127). In phase 2b clinical trials, however, no significant effect on NASH was observed in the patients treated with GR-MD-02 for 1 year compared to the placebo control (128).

In a mouse model of dilated cardiomyopathy, galectin-3 was upregulated by ∼40-fold in the heart, accompanied by dilation of cardiac chambers, reduced left ventricular ejection fraction, increased collagen content, and increased fibrosis. While galectin-3 gene knockout reduced these effects, MCP had no effect (129). In another study, however, MCP ameliorated cardiac dysfunction, reduced collagen deposition, and decreased myocardial injury, and galectin-3 expression in a rat model of heart failure (130). Belapectin was well tolerated by NASH patients but did not show anti-fibrotic effects in phase II clinical trial (131). Taken together, these studies imply that targeting just the carbohydrate-binding domain of galectin-3 is probably inadequate, as galectin-3 KO mice showed ameliorated fibrosis. Thus, most likely, both the N and C terminals of galectin-3 should simultaneously be targeted to combat fibrosis.

## Concluding remarks

Chimeric galectin-3 is a biological marker of active inflammation and advanced disease that could be clinically useful alone or in combination with collagen to serve as marker(s) and therapeutic target(s), based on the fact that collagen fragments are being used as biomarkers for fibrosis and inflammation-related diseases as a noninvasive method to investigate the disease progression. Their abundance and diversity, as well as several synthesis-related and lysis-related neoepitopes of each collagen type make it a very complicated story to analyze. Moreover, as the neoepitopes are detected in serum, it is hard to predict the site of fibrosis unless other markers and symptoms of the disease are investigated. For example, serum levels of PIIINP are enhanced in progression of the liver, heart, and lung disease, while some other neoepitopes are specific for a particular organ, *e.g.*, C6M and C3M are specific for liver fibrosis and PINP is specific for lung fibrosis (132). A detailed fingerprinting of various epitopes is required to be able to predict accurately the course of disease progression. Moreover, for accurate measurement of these markers, a specific antibody is crucial. Their levels can also be influenced by cellular uptake as some of the neoepitopes work as signaling molecules for cell surface receptors, *e.g.*, integrin receptors (reviewed by (9)).

Galectin-3 on the other hand belongs to a small family. Specific antibodies are present for different members of the family. Moreover, galectin-3, galectin-1, and galectin-9 are the only galectins related to fibrosis, with galectin-3 being the most thoroughly investigated. Galectin-3 is of particular interest because it can be used as a biomarker as well as therapeutic target for fibrosis as it is one of the earlier proteins released during neutrophil activation. The secreted protein is cleaved by MMPs in the same manner as collagens, and its cleavage has been proposed to be used as a diagnostic marker of MMP activity (15). A comparison of the 3-dimensional structure of galectin-1 and galectin-3 full-length and cleaved forms has been shown in Fig. 3. Cleaved galectin-3 retains some part of the N-terminal domain, making it different from galectin-1, which is made up only of the CRD. Specific antibodies are able to distinguish between the full-length and cleaved galectin-3 (15).

However, targeting galectin-3 has not been as successful as antifibrosis treatment, compared to the studies in KO mice. This could be because all the inhibitors used so far are designed specifically to target CRD. As presented in Table 1, both N-terminal and C-terminal domains of galectin-3 play a significant role in ligand binding. N-terminal domain is important for full biological activity of galectin-3. It facilitates proper folding of the protein, its multivalency, secretion, and its carbohydrate-binding properties. Flores-Ibarra *et al.* (133) reported formation of hairpin by the amino acids spanning Tyr^101^ in the N-terminal to Leu ^114^ in CRD. Miller *et al.* demonstrated that binding of polysaccharide rhamnogalacturonan to galectin-3 utilized two epitopes within the carbohydrate-binding domain, and one novel epitope within the first 40 amino acids of the N-terminal domain (134). To neutralize the microbicidal properties of galectin-3, protozoan parasites *Trypanosoma cruzi* and *Leishmania major* have developed molecular mechanisms to proteolytically cleave galecin-3 at its N terminus. The cleaved galectin-3 retained the carbohydrate-binding capacity but failed to induce the immune response (135, 136). In a recent report, Zhao *et al.* (137) reported that replacing the prolines in the N-terminal domain impaired all the classical cellular functions attributed to galectin-3. Mauris *et al.* demonstrated the requirement of a full-length protein for interaction with and clustering of CD147, an MMP-9 inducer in migrating epithelia (138). N-terminal domain of galectin-3 secreted by T cells upon T cell receptor engagement interacts with the proline-rich region of ALG-2–interacting protein X (Alix) in HIV infected cells facilitating HIV-1 budding (139). Bocker *et al.* found that Δ1-62 and Δ1-116 showed a 3- to 6-fold increased binding efficiency to asialofetuin compared to native galectin-3 when tagged with SNAP (140). Although galectin-3 enhances neutrophil activation by binding to microbial lipopolysaccharides *via* its carbohydrate recognition domain, N-terminal domain is also required, indicating dependence upon multivalency of galectin-3 (141, 142).

Considering the above, it may be worthwhile to target both N-terminal and C-terminal domains of galectin-3 to control fibrosis by small molecule inhibitors and/or galectin-3 domains-specific antibodies. DX-52-1, a semisynthetic derivative of quinocarmycin, and HUK-921, a complex synthetic molecule related to naphthyridinomycin family, are two compounds that were reported to bind galectin-3 outside of its carbohydrate domain (143) and exhibited antimigration and antiproliferation effects on cells and showed no competition with the agonist lactose. In addition, several noncarbohydrate small molecules have been designed and tested (reviewed by (144)) in an attempt to obtain more maintainable, reproducible, and effective galectin-3 inhibition, however, with limited success. The potential for galectin-3 as a therapeutic target in fibrosis remains a challenge together with the need to further unveil the molecular mechanism that regulates MMPs activities in the circulation. It is expected that due to the deleterious effects of fibrosis on organs’ functions, a new class of drugs/antibodies selectively and explicitly directed against the intact and cleaved galectin-3 will be available as a novel therapeutic modality, not too far away in the future.

## Conflict of interests

The authors declare that they have no conflict of interest with the contents of this article.
